# Brucellosis – laboratory workers’ nightmare come true: A case study

**DOI:** 10.4102/ajlm.v9i1.1114

**Published:** 2020-09-29

**Authors:** Lebogang Skosana, Farzana Ismail, Nontombi Mbelle, Mohamed Said

**Affiliations:** 1 Department of Medical Microbiology, Tshwane Academic Division, National Health Laboratory Services, Pretoria, South Africa; 2 Centre for Tuberculosis, National Institute of Communicable Diseases, Johannesburg, South Africa

**Keywords:** brucellosis, laboratory exposure, prophylaxis, public health, microbiology

## Abstract

**Introduction:**

*Brucella* spp. are rarely encountered organisms in the medical microbiology laboratory and, when encountered, can cause concern in laboratory workers. Laboratory personnel may in fact develop serious disease as a result of this exposure. This case highlights shortcomings in recognition of *Brucella* spp. from a patient presenting atypically as well as the follow-up and management of an infected patient.

**Case presentation:**

The patient was an 8-year-old boy from a rural area of South Africa who presented to an academic hospital with a bladder mass and history of enuresis in September 2016. *Brucella melitensis* was isolated from a blood culture submitted to the laboratory. The child was subsequently treated for brucellosis in November 2016.

**Management and outcome:**

The source of infection in the patient was traced to consumption of unpasteurised milk from a local farmer. The patient was treated with doxycycline 100 mg twice daily and rifampicin 600 mg daily for 6 weeks and completed treatment, however he was not followed up at our hospital. The laboratory personnel, however, did not handle the specimen as a Biosafety Level 3 pathogen as this organism is not commonly encountered; they were provided with prophylaxis for brucellosis (rifampicin and doxycycline).

**Conclusion:**

*Brucella* spp. is a dangerous pathogen, easily capable of causing significant exposure in an unsuspecting and unprepared laboratory. The case discusses the management of brucellosis in the infected patient as well as the management of laboratory exposure to *Brucella* spp. Our case also describes the public health response to a case of brucellosis.

## Introduction

Brucellosis is a zoonotic disease with a worldwide distribution that is caused by the *Brucella* genus, which are Gram-negative bacteria.^1^ There are approximately 500 000 brucellosis cases reported worldwide annually, however the figure may be much higher as the disease is under-reported, largely because of its non-specific signs and symptoms.^2^ Transmission to humans is through direct contact with animal reservoirs and/or through consumption of infected milk and milk products.^1,3^ The most common clinical presentation is undulant fever, malaise and arthralgia, after an incubation period ranging from four weeks to several months.^1^

Seven *Brucella* species are potentially pathogenic to humans and each species has a preferred animal host.^1,3,4^ These include *B. abortus* (cattle), *B. melitensis* (sheep, goats), *B. suis* (swine), *B. canis* (dogs), *B. ovis* (sheep), *B. ceti* (cetaceans), *B. pinnipedialis* (pinnipeds) and *B. inopinata* (unknown host).^1,3,4^ Brucellosis, particularly that caused by *B. melitensis*, is among the most frequently reported laboratory-acquired infections resulting from accidental or inadvertent exposure during aerosolisation procedures in the laboratory.^3^ This case aims to illustrate the holistic response required when a case of human brucellosis is encountered.

### Ethical considerations

This case study received ethical approval from the University of Pretoria Research Ethics Committee (Ethics Number: 412/2017). The patient’s mother was contacted and gave consent to publish by signing a consent form.

## Case presentation

The routine processing of blood culture specimens at the Tshwane Academic Division microbiology laboratory (Pretoria, South Africa) includes removal of positive blood culture bottles from the BacT/ALERT® (bioMérieux, Marcy-l’Étoile, France) system and performing a Gram stain, followed by direct sensitivity testing on those positive bottles. The bottle contents are then sub-cultured onto blood, chocolate and MacConkey agar plates routinely. Cultured plates are preliminarily examined after 10–12 hours of incubation and the chocolate plate is examined for any growth. If pure growth is noted, the plate is sent for identification using the Vitek® 2 automated system (bioMérieux, Marcy-l’Étoile France).

**FIGURE 1:**
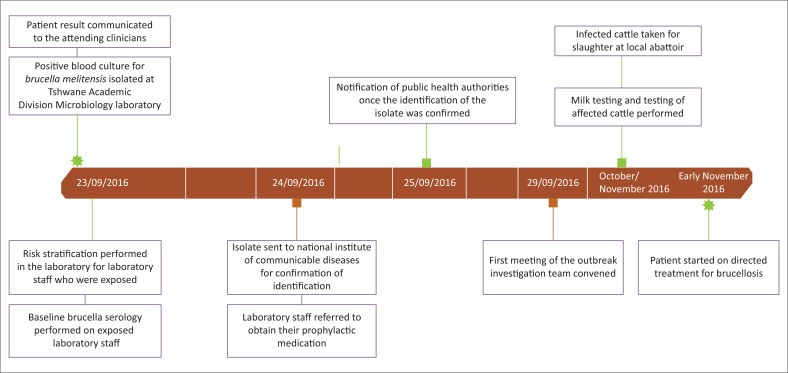
Significant timelines in the series of events related to the brucellosis case at Steve Biko hospital (Pretoria, South Africa) from September to November 2016.

In this case, the blood culture was taken from an 8-year-old boy from rural Mpumalanga, South Africa who was admitted to the Steve Biko Academic Hospital on 23 September 2016 and was being investigated for a bladder mass and a 2-year history of enuresis (Figure 1). For this case isolate, the chocolate plate had pure growth of fine grey colonies after 14 h of incubation. This isolate was sent for Vitek 2 for identification and was identified as *B. melitensis*. Once this identification was noted, all further processing was done in a biological safety cabinet class 2 using Biosafety Level 3 precautions, as recommended by the United States Centers for Disease Control and Prevention.^5^ A Gram stain from the culture plates revealed Gram-negative coccobacilli which were catalase and oxidase positive and indole negative which was in keeping with a preliminary identification of *Brucella* species. The positive *B. melitensis* culture was sent to the National Institute of Communicable Diseases in Sandringham, South Africa on 24 September 2016, where the identification of the organism was confirmed using matrix-assisted laser desorption/ionisation time of flight (MALDI-TOF) mass spectrometry. The instrument used in this case was the Vitek MS (bioMérieux, Marcy-l’Étoile, France), Instrument software version 1.5.0.4, MYLA version 4.5.1, Knowledge Base (Database) version 3.2 (bioMérieux, Marcy-l’Étoile, France).

## Management and outcomes

The blood culture was taken as part of ‘routine’ investigations as the child had no symptoms or clinical signs which may have led one to consider brucellosis as a possible differential diagnosis. The child was discharged once the blood samples were obtained. The outbreak response unit of the National Institute of Communicable Diseases was alerted to the case on 25 September 2016 and began further investigations and follow-up of affected individuals and possible contacts. It was discovered that the child’s family had been consuming unpasteurised cow’s milk from a local farmer for many years. This farmer’s cattle were tested in late October/early November 2016 using standard South African veterinary testing guidelines.^6^ Sixty-eight cows were tested and 13 were found to be positive for *B. melitensis*, using the Rose Bengal test, complement fixation test and serum agglutination test, all of which are serology-based tests. The affected cattle were then isolated, sent to an abattoir and slaughtered as stipulated in the control programme of brucellosis in animals of South Africa – *Animal Diseases Act* (Act No. 35 of 1984 ss. 9.1, 9.2, 11).^6^ The farmer was instructed to send his milk to another local farm for pasteurisation until this farmer was able to pasteurise effectively on his own farm.

The patient was treated with doxycycline 100 mg twice daily and rifampicin 600 mg daily for six weeks. He commenced treatment in the first week of November 2016. The treatment was initiated by the clinicians in his hometown and it was uncertain why he was started on the treatment six weeks following the initial blood culture result. Soon after commencing with treatment, the patient reported side effects which included abdominal pain and vomiting. These were managed symptomatically, and the patient was able to complete treatment. The affected patient’s family was tested serologically for brucellosis and were found to be negative. Prophylaxis was given to those family members who consented to receive it.

The United States Centers for Disease Control and Prevention’s brucellosis laboratory exposure risk stratification tool was used to screen for exposed staff and staff exposures were stratified as minimal risk, low risk and high risk using these guidelines.^5^ Twenty-one of seventy-two staff members were identified, who had either directly handled the specimen on an open bench or were within 1.5 metres of the person who handled the specimen on an open bench, thus rendering them at high risk for acquiring brucellosis.^5^ Baseline serology was performed on all potentially exposed staff members and standard prophylaxis was provided by the employer. This comprised of rifampicin 600 mg oral daily for three weeks as well as doxycycline 100 mg twice daily for three weeks.^5^ One staff member was pregnant (in her second trimester) at the time, and subsequently received trimethroprim-sulfamethoxazole (160/800 mg) twice daily for three weeks.^5^ Exposed staff were advised to undergo follow-up serology at 0, 6, 12, 18 and 24 weeks post-exposure.^5^ Weekly symptom watch forms were completed and daily fever self-checks were done for six weeks as recommended by the Centers for Disease Control and Prevention and the World Health Organization.^5,6^ None of the staff members reported any symptoms for the duration of the symptom check.

## Discussion

The transmission of *B. melitensis* to humans could be through direct contact with infected cattle or the products of abortion of the infected cattle through breaks in the skin as well as through the consumption of unpasteurised milk and milk products.^7^ Transmission can also occur as a result of laboratory exposure to the organism.^7^ *Brucella* spp. have an infective dose of 10–100 organisms and are often aerosolised during routine laboratory processing of microbiology specimens on an open bench.^5^ This processing could include performing routine tests on the bench, such as the catalase, oxidase test and indole tests, as well as performing antimicrobial susceptibility testing. These procedures could lead to exposure and possible infection with the organism and can be prevented by ensuring that all processing of *Brucella* spp. isolates is done in an appropriate biosafety cabinet employing Biosafety Level 3 precautions.^5,6^ Once our laboratory had a preliminary identification of *Brucella* spp., a decision was taken not to manipulate the culture further but to send the isolate to a reference lab with Biosafety Level 3 facilities for further processing. Even though the gold standard for definitive diagnosis of *Brucella* spp. is a positive culture, there is often a delay to final identification because of a number of factors: low index of suspicion, misidentification and unfamiliarity with the organism.

The process of identifying exposed individuals and informing them of their risk of developing brucellosis caused much panic among laboratory personnel, and counselling may have been inadequate when addressing fears in the workplace. Three staff members are known to have defaulted treatment because of a low perceived risk of acquiring brucellosis and also because of the intolerable side-effects of prophylactic drugs. Results of serological testing did not show any significant rise in antibody titres which could have suggested acute infection.

The laboratory had no standard operating procedure or policy for the management of possible exposure to a harmful organism before this case and were subsequently prompted to implement these.

Although *B. melitensis* mainly affects goats and sheep, cattle may also be infected as a result of indirect contact with goats and sheep.^8^ Furthermore, the systems used in this case may have misidentified the species of *Brucella.*^8^ The Vitek^®^ 2 automated system Gram-negative card can only identify the species *B. melitensis*, while the Vitek® MALDI-TOF MS (bioMérieux, Marcy-l’Étoile, France) was found to correctly identify *Brucella* spp. to the genus level with less correction for species-level identification.^8^ MALDI-TOF MS is a reliable method of species-level identification, provided that the *Brucella* spp. reference library used in the database is regularly updated.^9^

The strengths of this case were the rapid follow-up of exposed laboratory workers and their thorough assessment and management, which managed to contain any potential laboratory outbreak of brucellosis. A limitation of this study was the lack of follow-up on the patient’s clinical progress by the microbiology laboratory. This could be attributed to the outbreak investigation unit having taken over the follow-up of the case, as directed by the public health response protocol.

### Conclusion

In conclusion, this case highlights the occupational exposure to *B. melitensis* as well as the shortcomings associated with the laboratory management thereof. *Brucella melitensis* is a dangerous pathogen, easily capable of causing significant exposure in an unsuspecting and unprepared laboratory. Laboratories must ensure that staff are frequently reminded of risks of exposure to such organisms. Institutional policy documents should be updated and reviewed regularly, especially with regard to occupational hazards. Collaboration between different sectors (eg. agriculture, health) is needed to ensure adequate surveillance and control efforts.
