# Corrigendum: Brucellosis – Laboratory workers’ nightmare come true: A case study

**DOI:** 10.4102/ajlm.v10i1.1690

**Published:** 2021-11-22

**Authors:** Lebogang Skosana, Farzana Ismail, Nontombi Mbelle, Mohamed Said

**Affiliations:** 1 National Health Laboratory Services, Tshwane Academic Division, Pretoria, South Africa; 2 Department of Medical Microbiology, University of Pretoria, Pretoria, South Africa; 3 Centre for Tuberculosis, National Institute of Communicable Diseases, Johannesburg, South Africa

In the version of this article initially published, Skosana L, Ismail F, Mbelle N, Said M. Brucellosis – laboratory workers’ nightmare come true: A case study. Afr J Lab Med. 2020;9(1), a1114. https://doi.org/10.4102/ajlm.v9i1.1114, the authors’ second affiliation was omitted in the ‘Author’ and ‘Affiliation’ sections. The authors’ affiliations are now corrected as:

**Authors:**

Lebogang Skosana^1,2^ https://orcid.org/0000-0002-5684-9930

Farzana Ismail^2,3^ https://orcid.org/0000-0003-3070-9806

Nontombi Mbelle^1,2†^ https://orcid.org/0000-0001-8890-2663

Mohamed Said^1,2^ https://orcid.org/0000-0003-2610-0440

**Affiliations:**

^1^National Health Laboratory Services, Tshwane Academic Division, Pretoria, South Africa

^2^Department of Medical Microbiology, University of Pretoria, Pretoria, South Africa

^3^Centre for Tuberculosis, National Institute of Communicable Diseases, Johannesburg, South Africa.

This correction does not alter the study’s findings of significance or overall interpretation of the study’s results. The authors apologise for any inconvenience caused.

